# Effects of Different Surfactant Charges on the Formation of Gold Nanoparticles by the LASiS Method

**DOI:** 10.3390/ma14112937

**Published:** 2021-05-29

**Authors:** Muhammad Zulfajri, Wei-Jie Huang, Genin-Gary Huang, Hui-Fen Chen

**Affiliations:** 1 Department of Chemistry Education, Universitas Serambi Mekkah, Banda Aceh 23245, Indonesia; u106850002@kmu.edu.tw; 2 Department of Medicinal and Applied Chemistry, Kaohsiung Medical University, Kaohsiung 80708, Taiwan; u106550010@kmu.edu.tw (W.-J.H.); genin@kmu.edu.tw (G.-G.H.); 3 Department of Medical Research, Kaohsiung Medical University Hospital, Kaohsiung 80708, Taiwan; 4 Department of Chemistry, National Sun Yat-sen University, Kaohsiung 80424, Taiwan

**Keywords:** gold nanoparticles, laser ablation, LASiS method, morphology, surfactant, absorbance

## Abstract

The laser ablation synthesis in solution (LASiS) method has been widely utilized due to its significant prospects in laser microprocessing of nanomaterials. In this study, the LASiS method with the addition of different surfactant charges (cationic CTAB, nonionic TX-100, and anionic SDS) was used to produce Au NPs. An Nd:YAG laser system at 532 nm excitation with some synthetic parameters, including different laser fluences, ablation times, and surfactant concentrations was performed. The obtained Au NPs were characterized by UV-Vis spectroscopy, transmission electron microscopy, and zeta potential analyzer. The Au NPs exhibited the maximum absorption peak at around 520 nm for all samples. The color of Au NPs was changed from red to reddish by increasing the laser fluence. The surfactant charges also played different roles in the Au NPs’ growth during the synthesis process. The average sizes of Au NPs were found to be 8.5 nm, 5.5 nm, and 15.5 nm with the medium containing CTAB, TX-100, and SDS, respectively. Besides, the different surfactant charges induced different performances to protect Au NPs from agglomeration. Overall, the SDS and CTAB surfactants exhibited higher stability of the Au NPs compared to the Au NPs with TX-100 surfactant.

## 1. Introduction

Up to date, noble metal nanoparticles (NPs) especially silver (Ag) and gold (Au) have been widely explored and employed due to their distinct chemical, optical, and electronic properties [1]. Particularly, different numbers of Au atoms in small Au NPs generate certain chemical and physical characteristics [2]. Due to their unique size-dependent properties, Au NPs are very useful for catalysis [3], chemo/biosensors [4,5], electrochemical sensors [6], electronic devices [7], solar cells [8], drug delivery [9], cancer therapy [9], and antibacterial activity [10]. Accordingly, it is necessary to obtain small Au NPs with controlled particle sizes.

The synthesis of metal NPs in a solution is one of the prominent subjects in the physics and chemistry of nanomaterials [11]. The conventional methods including mechanical grinding or chemical methods such as the sol-gel process have drawbacks correlated with the purity and variation of produced NPs [12]. A chemical reduction of Au salts [13], gas-phase deposition [14], and radiolytic reduction [15,16], can produce the Au NPs with different shapes and sizes. However, these methods have some restrictions such as trouble to control the size and shape of NPs, poor reproducibility, difficult separation, and complicated surface functionalization of NPs. From the preparation viewpoint, a chemical reduction of metal ions is widely used for producing NPs in solution [17]. On the other hand, scientists still explore the safe and facile methods for the synthesis of Au NPs because of the harmful effects of reducing agents, organic solvents, and poisonous reagents employed in the synthesis of Au NPs for the human health and environment [18]. Laser ablation is an alternative method to solve the limitations of the above-mentioned conventional methods.

The laser ablation synthesis in solution (LASiS) method is one of the promising eco-friendly methods for producing metal NPs [19]. LASiS method becomes more popular because of its fast, facile, cheap, and green production process of NPs in water or organic solvents [20,21]. It has important benefits for biological applications where the NPs’ surface is not contaminated with residual ions deriving from reactants [22]. It is also used as the adjustable and eco-friendly method to obtain NPs with the desirable structure without impurities [23]. However, the NPs prepared using this method in water commonly suffer from inhomogeneous particle sizes with an apparent aggregation [24]. The interaction between the pulsed laser and metal NPs induces some particle aggregates and a wide size distribution via the fragmentation/melting process [25]. Meanwhile, a surfactant, used as a capping agent, commonly plays an important role to control the stability and size of the NPs. The particle–particle interaction will be much higher without the surfactant, promoting the instability of Au NPs. The surfactant molecules adsorb on the NP surfaces to maintain the dispersion of NPs in the liquid phase. The addition of surfactants that cover the NPs during the ablation process improves the size uniformity and prevents the coalescence of the NPs [26]. The surfactant capping and the NPs’ surface charge are closely related to the stability of the NPs [27]. Therefore, the stability and size distribution of the NPs highly depends on the type and amount of the employed surfactant [28].

Although the influences of the surfactants on the size distribution and stability of the laser-ablated metal NPs have been entirely explored [11,24,26,29,30,31,32], very few studies were reported for the comparative study of the effects of surfactants with different charges along with the adjustment of different synthesis parameters. Considering the tremendous advantages of Au NPs and the versatility of the LASiS method, the Au NPs were produced using the LASiS method at 532 nm laser-focused on an Au plate in the present study. Different surfactant charges (cationic CTAB, nonionic TX-100, and anionic SDS) were added to the media as capping agents. Encouraging results were obtained for expectant applications by adjusting several parameters including the laser fluence, ablation time, and surfactant concentration to control the growth of Au NPs. Besides, the average diameter size and stability of obtained Au NPs were also measured. The characteristics of the obtained Au NPs using different surfactant charges were compared.

## 2. Materials and Methods

### 2.1. Materials and Chemicals

An Au plate (99.99%) and a 20 mL pyrex vial bottle as a container were used in this study. Cetyltrimethylammonium bromide (CTAB, 98%) was obtained from Alfa Aesar (Ward Hill, MA, USA). Triton X-100 (TX-100, 99%) was used as received from Sigma-Aldrich (St. Louis, MO, USA). Dodecylsulfuric acid sodium salt (SDS, 99%) was obtained from Sigma-Aldrich. Doubled distilled water was utilized during the experiments.

### 2.2. Preparation of Au Plate

The Au plate was cut in advance with a ratio of 1.2 cm × 1.2 cm and a thickness of 0.1 cm. The prepared Au plate was washed twice after being repeatedly polished with water-resistant coarse sandpaper (cc-180) and fine sandpaper (cc-320). Subsequently, the clean Au plate was wiped and then placed in the bottom of the container immediately.

### 2.3. Installation of Laser Beam Path

**Figure 1:**
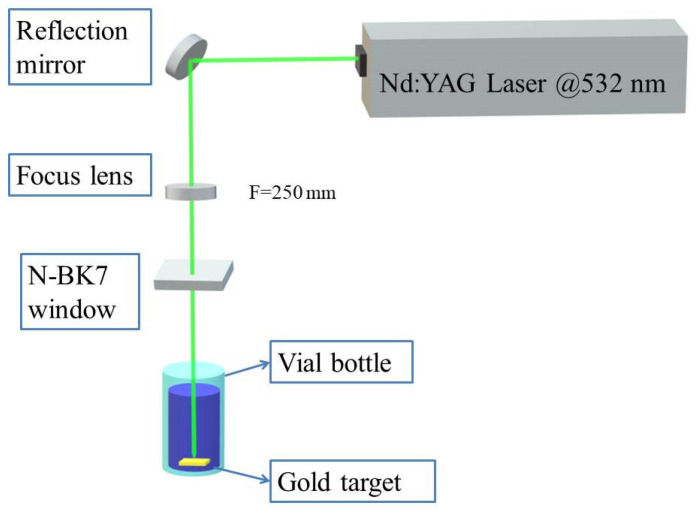
A schematic diagram of the LASiS method procedure for the synthesis of Au NPs.

The focus lens at a distance of 250 mm from the Au plate position was set. After the laser beam passed through the focusing lens, the diameter was reduced from 7 mm to 0.5 mm. It was confirmed that the optical path had no other interference. As depicted in Figure 1, the Au plate was placed in the bottom of the synthesis container containing the aqueous solution of surfactant.

### 2.4. Production of Au NPs

Au NPs were produced by the LASiS method in the aqueous solutions of surfactants. The Au plate was irradiated with an output of the second harmonic (532 nm) of Continuum SLI-10 Surelite ND:YAG laser (Loveland, CO, USA)operating at a repetition rate of 10 Hz and 10 ns pulse width (FWHM). The laser beam was focused on the Au plate center. The Au plate was normally moved to utilize a motorized X-Y stage at a constant speed of 5 mm/min to prevent the creation of craters causing a uniform surface ablation.

Furthermore, the laser fluence, ablation time, and surfactant concentration were evaluated. For the effect of the laser fluence, 5.7 mL of H_2_O and 0.3 mL of 200 mM surfactants were inserted into a container. The laser fluence was set at 0.1, 0.2, and 0.4 J cm^−2^. The laser beam focused at the Au plate center was aligned with 20 min of ablation time. For the effect of the ablation time, the laser fluence was adjusted at 0.4 J cm^−2^. Different laser ablation times were carried out for 20 min until 100 min. For the effect of surfactant concentration, 5.7, 5.97, and 5.997 mL of H_2_O and 0.3, 0.03, and 0.003 mL of 200 mM surfactants (final concentrations: 10, 1.0, and 0.1 mM) were used. The laser fluence and ablation time were adjusted at 0.4 J cm^−2^ and 40 min, respectively.

### 2.5. Characterizations

The UV-Vis spectra of Au NPs were measured using a V-730 UV-Visible Spectrophotometer from JASCO (Easton, MD, USA). An amount of 3.0 mL of Au NPs was placed inside the cuvette cell with an optical path length of 1 cm. The TEM images were captured utilizing a JEOL JEM-2100 TEM microscope (Tokyo, Japan). The Au NPs solution was sonicated for 30 min and placed a drop of sample onto a Cu mesh coated with an amorphous carbon film, followed by drying it in a vacuum desiccator for a day before measurement. The ImageJ Launcher (Version 1.43.67, Broken Symmetry Software, Bethesda, MD, USA, 2006) was utilized for an image processing software to analyze the size of the Au NPs. Zeta potential values of Au NPs were recorded using an ELSZ-2000 Instrument (Otsuka, Japan). A centrifuge 5424 from Eppendorf was used to separate the samples from the biggest particles resulting mostly from ablation debris during the synthesis.

## 3. Results and Discussion

### 3.1. Effect of Laser Fluence

**Figure 2:**
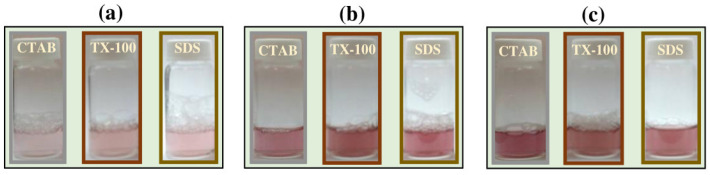
The solution color of the Au NPs using different surfactants at (**a**) 0.1 J cm^−2^, (**b**) 0.2 J cm^−2^, and (**c**) 0.4 J cm^−2^. The ablation time was 20 min and the surfactant concentration was 10 mM.

The effect of laser fluence was examined for 20 min of ablation time and 10 mM surfactant concentration. A visible pink color of the solution was appeared several times after starting the ablation, indicating the formation of Au NPs. This color is related to the surface plasmon resonance (SPR) absorption of Au NPs in liquid [33]. The pink color was viewed after 20 min of the ablation time. The color was increased to reddish by enhancing the laser fluence from 0.1 to 0.4 J cm^−2^ (Figure 2), indicating the increase in Au NPs’ concentration. Besides, the bubbles were generated in the solution due to the liquid evaporation during the ablation process. The bubble formation cannot be prevented as a consequence of liquid vaporization under intense laser radiation on the Au plate surface. After some pulsed laser irradiated on the Au plate, the vapor bubbles were broken up into small ones which can significantly scatter the laser beam and in turn attenuate the laser intensity at the work surface and disturb the ablation performance [34]. The loss of laser energy during the laser ablation process is subject to the light absorption of liquid, dynamic refraction of the laser beam at the air-liquid interface, and the beam scattering caused by vapor bubbles at the laser-heated area. However, the control of bubble size and lifetime would be an approach to decrease the bubble disturbances to the the laser beam [34].

**Figure 3:**
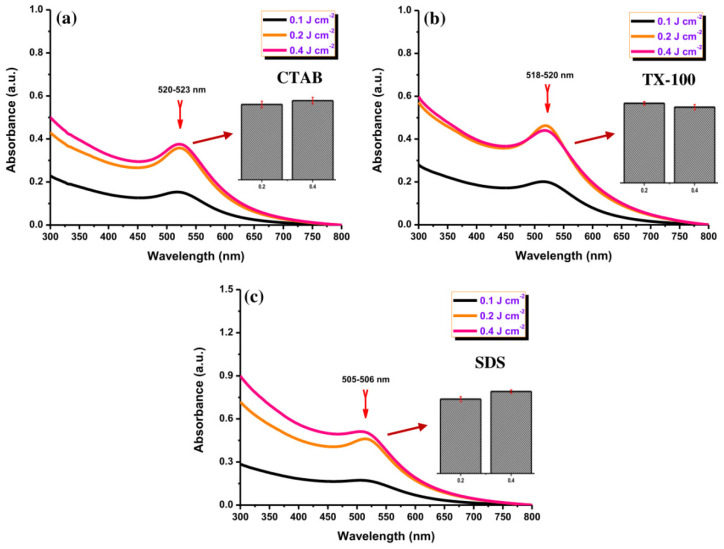
The UV-Vis absorption spectra of Au NPs using using (**a**) CTAB, (**b**) TX-100, and (**c**) SDS (10 mM) with different laser fluences (0.1, 0.2, and 0.4 J cm^−J^). Inset: the maximum absorbance comparison of Au NPs with 0.2 and 0.4 J cm^−2^ for all surfactants. The ablation time was 20 min.

The UV-Vis absorption spectra of the Au NPs were measured after preparation with different laser fluences (Figure 3). The red color is correlated with the maximum absorption peak at ~520 nm for CTAB and TX-100 (Figure 3a,b) and ~505 nm for SDS (Figure 3c), indicating the formation of Au NPs. No red-shift was observed for all samples with different laser fluences, indicating no significant change in the NPs. The production efficiency of Au NPs was low with a low laser fluence (0.1 J cm^−2^) for all surfactants. Meanwhile, it can be seen clearly that the absorption peak of Au NPs at 0.2 J cm^−2^ and 0.4 J cm^−2^ was almost closer (Inset Figure 3). So that 0.4 J cm^−2^ was selected for further experiments.

**Figure 4:**
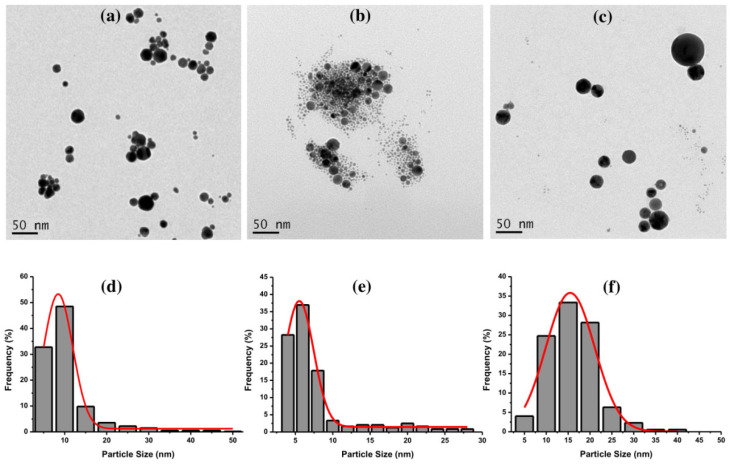
The TEM images of Au NPs using (**a**) CTAB, (**b**) TX-100, and (**c**) SDS, and their size distributions (**d**) CTAB, (**e**) TX-100, and (**f**) SDS with the laser fluence of 0.4 J cm^−2^. The ablation time was 20 min, and the surfactant concentration was 10 mM.

Furthermore, TEM images of Au NPs with 0.4 J cm^−2^ of laser fluence were captured to obtain the particle size and shape. Figure 4 depicts the TEM images of Au NPs using CTAB (Figure 4a), TX-100 (Figure 4b), and SDS (Figure 4c). The Au NPs using CTAB have a spherical shape with an average size of 8.5 nm. Meanwhile, the Au NPs using TX-100 have a narrow average size of 5.5. nm with spherical shape. The Au NPs’ spherical shapes using SDS appeared to clearly separate from each other compared to the spherical shapes of Au NPs using CTAB and TX-100. Overall, the average sizes of Au NPs were quite small with uniform particle shapes. The size distribution of all Au NPs with different surfactants can be seen in Figure 4d–f.

### 3.2. Effect of Laser Ablation Time

**Figure 5:**
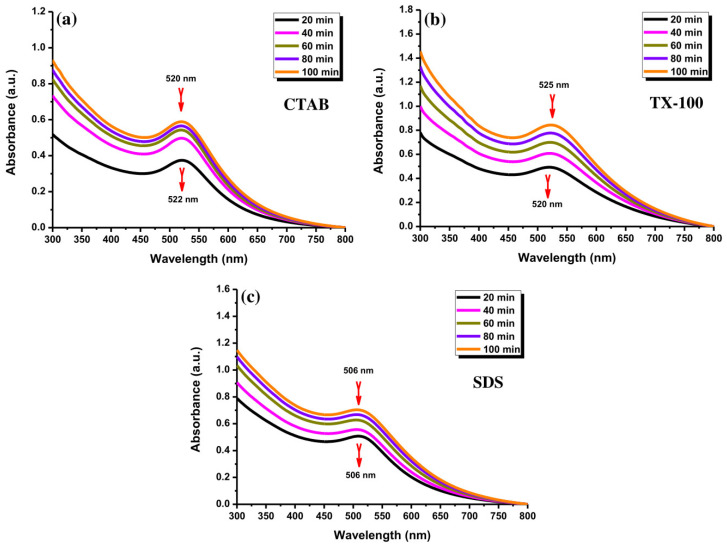
The UV-Vis absorption spectra of Au NPs using (**a**) CTAB, (**b**) TX-100, and (**c**) SDS (10 mM) with different laser ablation times (20, 40, 60, 80, and 100 min) at laser fluence of 0.4 J cm^−2^.

The effect of different laser ablation times from 20 to 100 min was examined for the preparation of Au NPs. As depicted in Figure 5a, the Au NPs with CTAB exhibited a significant increase in their absorption peak with the extension of the ablation time without a red-shift behavior, indicating the enhancement of Au NPs’ concentration. The absorption peak of Au NPs using TX-100 increased with increasing the ablation time and a slight red-shift of about 5 nm (Figure 5b). The red-shift indicates the growth in particle sizes of Au NPs. Besides, the absorption peak of Au NPs using SDS also increased by lengthening the ablation time (Figure 5c). No red-shift behavior was observed with maintaining the absorption peak center position. The Au NPs with CTAB and SDS possessed lower absorbance compared to the Au NPs with TX-100 while increasing the ablation time. Overall, the lengthening of ablation time would gradually increase the concentration of Au NPs.

**Figure 6:**
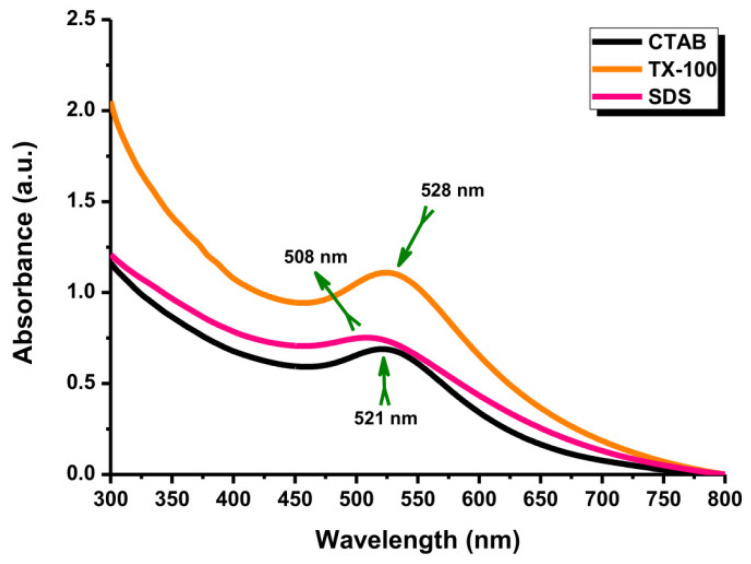
The UV-Vis absorption spectra of Au NPs with different surfactants. Conditions: Surfactant concentration: 10 mM, laser fluence: 0.4 J cm^−2^, and ablation time: 200 min.

**Figure 7:**
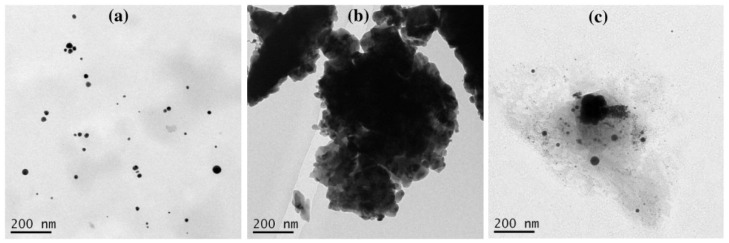
The TEM images of Au NPs using (**a**) CTAB, (**b**) TX-100, and (**c**) SDS with ablation time of 200 min and the laser fluence of 0.4 J cm^−2^. The surfactant concentration was 10 mM.

In the LASiS method, it may cause multiple erosions on the Au NPs surface if the laser ablation time is too long, which perturbs the interface activity between Au NPs and surfactants and reduces the protective effect of the surfactant as a stabilizer. According to Figure 6, the Au NP coating with the surfactants at a long ablation time (200 min) might exhibit a significant change in UV–Vis absorption peak intensity where the agglomeration would be formed. For instance, the Au NPs with TX-100 might be agglomerated seriously because its protective effect was reduced. The agglomeration of Au NPs with TX-100 happened (Figure 7a) compared to Au NPs with CTAB (Figure 7a) and SDS (Figure 7c). The spherical shape was almost incomplete, and the probability of producing large particles was extremely high. TX-100 with neutral surface charge compared to SDS (negative charge) and CTAB (positive charge) exhibited lower stability of Au NPs produced in a long ablation time. Although TX-100 can increase the viscosity of the solution and inhibit the growth of large bubbles/particles, the extension of the ablation time will weaken the influence of the above two effects [35].

### 3.3. Effect of Surfactant Concentration

**Figure 8:**
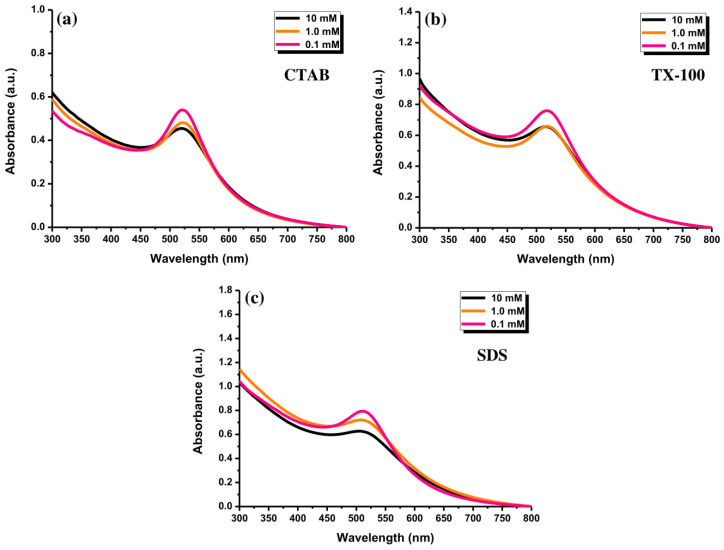
The UV-Vis absorption spectra of Au NPs after ablating for 40 min with (**a**) CTAB, (**b**) TX-100, and (**c**) SDS, at the concentrations of 0.1, 1.0, and 10 mM and laser fluence of 0.4 J cm^−2^.

Au NPs with different CTAB concentrations exhibited almost similar absorption peak behavior at ~520 nm (Figure 8a). Forty minutes of ablation time was set for the experiment. Besides, Figure 8b exhibits a similar position of the absorption peaks (~520 nm) of Au NPs with TX-100 at different concentrations. The absorption peak of Au NPs using different SDS concentrations have almost in a similar position (Figure 8c). The absorption peaks of the samples were centered at ~505 nm. The absorption peak center of Au NPs with 0.1 mM surfactants reached a higher intensity compared to two other surfactant concentrations. When the surfactant concentrations were at 1.0 mM and 10 mM, the absorption peaks of both concentrations were almost close. The different concentrations of surfactants might not affect the change of the absorption peak positions of Au NPs, indicating no perturbation of the morphology patterns of Au NPs. It is believed that the morphology of the produced Au NPs was almost similar. The critical micelle concentrations (CMCs) of CTAB, TX-100, and SDS were 0.91 mM, 0.26 mM, and 8.6 mM, respectively [36]. For the surfactant concentrations above the CMC, stable NPs were usually formed [37]. A smaller amount of the surfactant does not perfectly cover the entire surface of the NPs, which can cause coalescence of the NPs or an uncontrolled increase in particle size [38].

### 3.4. Stability of Au NPs

#### 3.4.1. Storage Time

**Figure 9:**
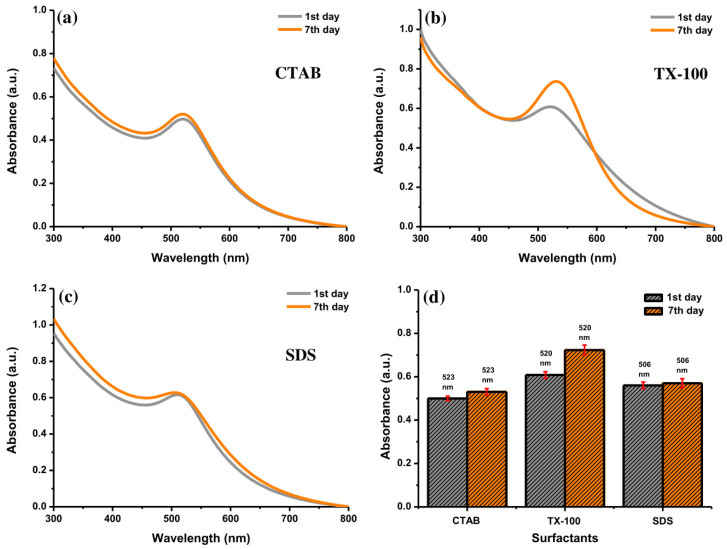
The UV-Vis absorption spectra of Au NPs with (**a**) CTAB, (**b**) TX-100, and (**c**) SDS after preparation and after storing for 7 days, and (**d**) their comparison at specific absorption peak of Au NPs with different surfactants. Conditions: surfactant concentration = 10 mM, laser fluence = 0.4 J cm^−2^, and ablation time = 40 min.

**Figure 10:**
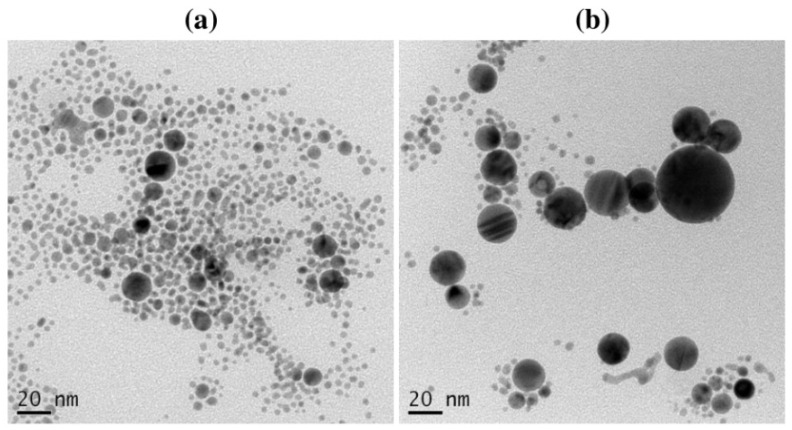
The TEM images of Au NPs with TX-100 after (**a**) preparation and (**b**) storing for 7 days. Conditions: TX-100 concentration = 10 mM, laser fluence = 0.4 J cm^−2^, and ablation time = 40 min.

The UV-Vis absorption spectra of Au NPs were observed to determine the effect of storage time. The decay of the NPs placed for a long time may be established. The stability of Au NPs with 10 mM of surfactants was evaluated. As shown in Figure 9a, the absorbance of Au NPs using CTAB at 523 nm increased insignificantly after storing for one week. Conversely, the absorption peak of Au NPs with TX-100 at 520 nm increased significantly with a slight red-shift character about 10 nm (Figure 9b). It can be inferred that the effect of TX-100 was getting weaker, but it has not been lost for all. Some particles have a bigger size after storing for 7 days as shown in Figure 10. The stability of Au NPs induced by TX-100 is mainly related to the steric stabilization, which can be linked to the reorganization of the surface structure. The change in the stability of Au NPs using TX-100 after a long storage time can occur due to the the weak adsorption of the TX-100 molecules onto the Au NP surface and subsequently would affect the particle size of Au NPs. The weaker stability is due to the structural nature of the surfactant molecules reflected in the hydrophilic–lipophilic balance (HLB) value of nonionic surfactants (0–20) [39]. The TX-100 has the HLB value of 13.5 [40]. Due to a higher HLB value of TX-100, the surfactant tends to exhibit less effective stability and tends to become hydrophilic. Less stable Au NPs would increase the particle–particle interactions affecting the particle size of Au NPs. In addition, the absorption peak of Au NPs with SDS at 506 nm was almost similar (Figure 9c). Accordingly, higher stability was observed for Au NPs with SDS and CTAB was observed compared to TX-100 after storing for one week (Figure 9d). Therefore, the Au NPs can be stored at least for one week.

#### 3.4.2. Zeta Potential

**Figure 11:**
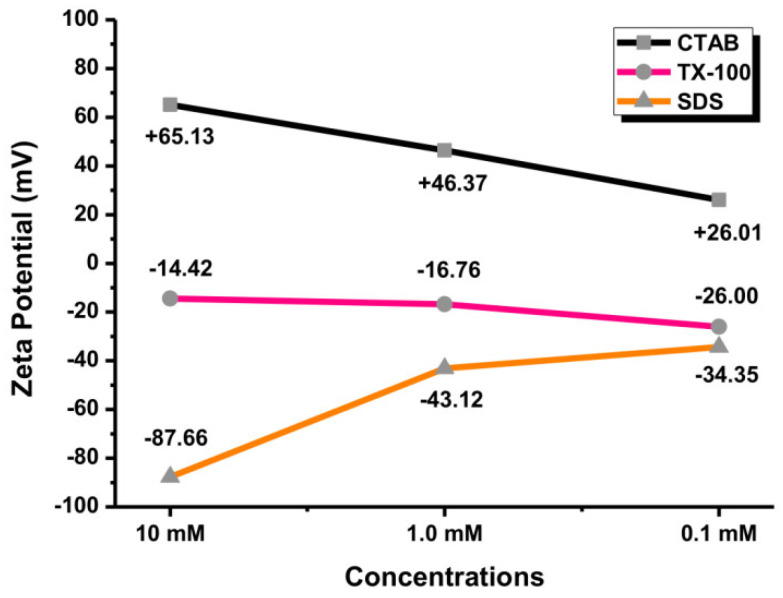
The zeta potential values of Au NPs with different concentrations of surfactants (0.1, 1.0, and 10 mM).

The stability of colloidal NPs can be assessed by measuring their zeta potential (ZP) value. The ZP values of ±0–10 mV indicate an unstable colloid. Meanwhile, the ZP values of ±10–20 mV, ±20–30 mV, and ˃±30 mV indicate the low, moderate, and high stability of colloid, respectively [41,42]. Amounts of 0.1, 1.0, and 10 mM of surfactants were used for the measurement. By measuring their surface charges, it can be ensured that the Au NPs were covered by the surfactants. The Au NPs with CTAB as a cationic surfactant exhibited a highly positive surface charge. Meanwhile, SDS as an anionic surfactant exhibited a highly negative surface charge of Au NPs. TX-100 is a nonionic surfactant so that the surface charge of Au NPs was in the low and moderate stability range (<30 mV). As shown in Figure 11, the ZP value of Au NPs with 0.1 mM CTAB was +26.01 mV, reflecting the moderate stability of Au NPs. The surface charges of Au NPs with 1.0 and 10 mM CTAB were greater than +30 mV. The surface charge of Au NPs with 0.1 SDS was −34.35 mV, indicating highly stable NPs. Overall, the surface charge of Au NPs with TX-100 was in the relatively and moderately stable range of NPs (<±30 mV). Therefore, the Au NPs with SDS and CTAB showed higher stability of colloidal particles compared to TX-100. The agglomeration tendency of Au NPs in TX-100 is high due to its low zeta potential value [43]. However, the surface charge is not an absolute requirement for colloid stability. Other parameters can be adjusted to promote the stability of Au NPs such as pH, temperature, pressure, storage time, salinity, particle size, and environmental conditions [44,45].

### 3.5. Possible Formation Mechanism of Au NPs

For the possible formation of Au NPs from an Au plate using a laser ablation [11,24,27], a core made of a small number of Au atoms formed accidentally by density fluctuation in a cloud which continues to grow until Au atoms are consumed almost completely in the vicinity. After the focused laser ablation to the Au plate, a dense cloud of Au atoms (plum) is built over the laser spot of the Au plate. The particle thus produced is the embryonic particle. The Au NPs are formed through the rapid formation of an embryonic Au particle. The Au NPs with relatively uniform size are formed in the laser ablation process. However, the slow NPs growth is terminated by coating the NPs surface with the surfactant molecules. Surfactants added into the solution will decrease the contact angle of NPs. A decrease in contact angle may increase the dispersibility of Au NPs [43]. The surface charge is controlled by nature and the charge of ions adsorbed on the NPs to stabilize the NPs. The interaction between the Au NPs and surfactants was found to be dependent on the surfactant charges which affected the stability and growth rate of Au NPs.

The SDS and CTAB micelles through the electrostatic repulsion and TX-100 through the spatial repulsion prevent the particles from sticking together. As stated by Chen and Yeh [24], the stability of Au NPs with SDS is correlated with whether the SDS double layers on the NPs surface or not. The formation of double layers on the NPs leads to a longer colloidal lifetime. The double layers constitute hydrophilic –SO_4_^−^ groups toward the positively charged Au and with hydrophobic hydrocarbon chains directed outward to form the first layer. Conversely, the second layers oppositely orient the SDS molecules, resulting in the interpenetration of the surfactant hydrophobic tails between the two layers with –SO_4_^−^ headed outward. On the other hand, instead of the N(CH_3_)_3_^+^ group as the hydrophilic end, it is most likely that the CTAB’s hydrocarbon chains surround the Au NPs owing to electrostatic interaction. The laser irradiation of Au plate in TX-100 aqueous solution forms bare NPs and/or partially coated NPs by TX-100 [46]. TX-100 attains a surface coverage at which aggregation of NPs is inhibited and the NPs can be dispersed stably in the aqueous solution.

### 3.6. Comparative Effects of Au NPs by Different Parameters

The addition of different surfactants has a significant impact on the production of Au NPs using the LASiS method. Using CTAB, a low fluence of 0.1 J cm^−2^ produced the Au NPs with a low concentration. By changing the ablation time, the absorbance of Au NPs was gradually increased. The absorption peak of the Au NPs with 20 min ablation time was low. The extension of the ablation time increased the particle concentration of Au NPs. An amount of 10 mM CTAB produced the Au NPs with efficient absorbance. By controlling the CTAB concentration, the deformation of the Au NPs can be effectively suppressed. Meantime, the Au NPs with TX-100 as nonionic surfactant would not produce a red-shift of the absorption peak position by increasing the laser fluence. The extension of ablation time might cause the agglomeration of Au NPs to form a sheet-like structure. Next, production of the Au NPs with SDS as an anionic surfactant, at 0.1 J cm^−2^ laser fluence, was low. Meanwhile, the absorption peak of Au NPs with 10 mM SDS increased with increasing the laser ablation time. The Au NPs using SDS possessed no obvious change in the absorption peak position at 0.4 J cm^−2^ with different SDS concentrations. According to the zeta potential analysis, the stability of Au NPs using CTAB and SDS for all concentrations was better than TX-100. Based on the findings, the formation of Au NPs by the LASiS method can be achieved with an optimal laser fluence of 0.4 J cm^−2^, ablation time of 40 min, and surfactant concentration of 10 mM.

## 4. Conclusions

Herein, Au NPs were produced by a LASiS method with different surfactant charges (CTAB, TX-100, and SDS). The type of surfactants affected the structure, size, and stability of Au NPs. Different laser fluences, ablation times, and surfactant concentrations played a significant role in producing spherical and small dispersed Au NPs. The surfactants were added to avoid the agglomeration of Au NPs. Overall, the Au NPs have a spherical shape with a small size. The increase in the ablation time would increase the absorbance of Au NPs, indicating the increase in Au NPs’ concentration. The absorption peak position of Au NPs using TX-100 slightly red-shifted by lengthening the ablation time. A long ablation time might reduce the protective effect of TX-100. This result might require more attention in future synthesis. Based on the zeta potential analysis, the Au NPs with a ZP value lower than 30 mV have lower stability. Au NPs with SDS and CTAB have higher stability than TX-100. The production of Au NPs using the LASiS method can be controlled more effectively. This study confirms that the LASiS method is a suitable and competitive way to produce Au NPs with different surfactant charges.

## Data Availability

Data sharing is not applicable.
